# New Staphylinidae (Coleoptera) records with new collection data from New Brunswick, Canada: Pselaphinae

**DOI:** 10.3897/zookeys.186.2505

**Published:** 2012-04-26

**Authors:** Reginald P. Webster, Donald S. Chandler, Jon D. Sweeney, Ian DeMerchant

**Affiliations:** 1 Natural Resources Canada, Canadian Forest Service - Atlantic Forestry Centre, 1350 Regent St., P.O. Box 4000, Fredericton, NB, Canada E3B 5P7; 2 Department of Biological Sciences, University of New Hampshire, Durham, NH, USA 03824

**Keywords:** Staphylinidae, Pselaphinae, new records, Canada, New Brunswick

## Abstract

Twenty species of Pselaphinae are newly recorded from New Brunswick, Canada. This brings the total number of species known from the province to 36. Thirteen of these species are newly recorded for the Maritime provinces of Canada. *Dalmosella tenuis* Casey and *Brachygluta luniger* (LeConte) are newly recorded for Canada. Collection and habitat data are presented for these species.

## Introduction

This paper treats new Staphylinidae records from New Brunswick from the subfamily Pselaphinae. Taxonomically, the North American species of Pselaphinae are fairly well known. Most species in the Maritime provinces (New Brunswick, Nova Scotia, Prince Edward Island) of eastern Canada can be determined using keys and descriptions in Grigarick and Schuster (1980; *Bibloporus*, *Dalmosella*), Wagner (1975), Chandler (1986; *Euplectus*, *Pycnoplectus*), Carlton and Chandler (1994; *Ramecia*), Chandler (1990; *Bibloplectus*, *Trimioplectus*), Grigarick and Schuster (1971; *Actiastes*, *Actium*), Chandler (1986, 1993; *Actizona*), Chandler (1991; *Lucifotychus*), Chandler (1989) and Carlton (2003; *Reichenbachia*), Park (1947; *Batrisodes*), Fall (1927; *Rybaxis*), Carlton (1989; *Eutrichites*), Park (1958; *Decarthron*), Casey (1894; *Brachygluta*, *Pselaphus*), and Chandler (1999; *Tyrus*). Chandler (1997) summarized information on biology and taxonomy of the North American species and noted availability of keys to genera in his catalog.

Species of Pselaphinae from eastern Canada are found in moss, grass, and leaf litter in marshes, bogs, and along stream margins, the intertidal zone of salt marshes, forest floor litter, in rotten logs, under bark of dead trees and logs, in tree holes, and in ant nests (Park 1947, 1949; Park et al. 1950; Reichle 1969; Wagner 1975; Chandler 1987, 1989, 1991, 1993, 1997). Adults are predators of mites, Diptera larvae, earthworms, ant eggs and larvae, collembolans, and other small invertebrates (Park 1929, 1932a, 1932b, 1947; Grigarick and Schuster 1971; Chandler 1997, 2000). Some species may be important as indicators of old-growth forests (Chandler 1987; Carlton and Chandler 1994).

Over 710 species of Pselaphinae are known from North America (Chandler 1997). Eighty-five species were reported from Canada by Davies (1991), with 15 of these recorded from New Brunswick. Majka and Ogden (2006) reported *Brachygluta abdominalis* (Aubé) new to NS and NB, and to Canada. Here, we report 21 species new to New Brunswick, including two species new to Canada.

## Methods and conventions

The following records are based on specimens collected during a general survey by the first author to document the Coleoptera fauna of New Brunswick, and from the by-catch of samples obtained during a study to develop a general attractant for the detection of invasive species of Cerambycidae.

### Collection methods

Various collection methods were employed to collect the species reported in this study. Details are outlined in Campbell (1973) and Webster et al. (2009, Appendix). See Webster et al. (2012) for details of the methods used for deployment of Lindgren 12-funnel traps and sample collection. A description of the habitat was recorded for all specimens collected during this survey. Locality and habitat data are presented exactly as on labels for each record. This information, as well as additional collecting notes, is summarized and discussed in the collection and habitat data section for each species.

### Specimen preparation

Males of *Actiaste*s, *Euplectus*, and *Pycnoplectus* were dissected in order to confirm their identity. The genital structures were dehydrated in absolute alcohol and either mounted in Canada balsam on celluloid microslides and pinned with the specimens from which they originated, or glued onto points with the specimens.

### Distribution

Distribution maps, created using ArcMap and ArcGIS, are presented for each species in New Brunswick. Every species treated has its currently known distribution in Canada and Alaska indicated, using standard two-letter abbreviations for the states, provinces, and territories. New records for New Brunswick are indicated in bold under Distribution, which covers Canada and Alaska. The following abbreviations are used in the text:

**Table:** 

**AK**	Alaska	**MB**	Manitoba
**YT**	Yukon Territory	**ON**	Ontario
**NT**	Northwest Territories	**QC**	Quebec
**NU**	Nunavut	**NB**	New Brunswick
**BC**	British Columbia	**PE**	Prince Edward Island
**AB**	Alberta	**NS**	Nova Scotia
**SK**	Saskatchewan	**NF & LB**	Newfoundland and Labrador

Acronyms of collections examined or where specimens referred to in this study reside are as follows:

**AFC** Atlantic Forestry Centre, Natural Resources Canada, Canadian Forest Service, Fredericton, New Brunswick, Canada

**CNC** Canadian National Collection of Insects, Arachnids and Nematodes, Agriculture and Agri-Food Canada, Ottawa, Ontario, Canada

**NBM** New Brunswick Museum, Saint John, New Brunswick, Canada

**RWC** Reginald P. Webster Collection, Charters Settlement, New Brunswick, Canada

## Results

Twenty species of Pselaphinae are newly recorded from New Brunswick. This brings the total number of species known from the province to 36. Thirteen of these species are newly recorded for the Maritime provinces of Canada. Two are newly recorded for Canada.

## Species accounts

All records below are species newly recorded for New Brunswick, Canada. Species followed by ** are newly recorded from the Maritime provinces of Canada. A list of species of Pselaphinae currently known from New Brunswick is given in Table 1.

**Table 1.** Species of Pselaphinae (Staphylinidae) recorded from New Brunswick, Canada.

**Subfamily Pselaphinae Latreille**
**Supertribe Euplectitae Streubel**
**Tribe Euplectini Streubel**
*Actiastes foveicollis* (LeConte)**
*Actiastes globiferum* (LeConte)**
*Bibloplectus integer* (LeConte)
*Bibloporus bicanalis* (Casey)*
*Dalmosella tenuis* Casey***
*Euplectus acomanus* Casey**
*Euplectus confluens* LeConte**
*Euplectus duryi* Casey
*Euplectus elongatus* Brendel*
*Pycnoplectus linearis* (LeConte)*
*Ramecia crinita* (Brendel)*
*Trimioplectus obsoletus* Brendel**
**Supertribe Batrisitae Reitter**
**Tribe Batrisini Reitter**
*Batrisodes frontalis* (LeConte)**
*Batrisodes lineaticollis* (Aubé)
*Batrisodes riparius* (Say)**
*Batrisdoes scabriceps* (LeConte)**
**Supertribe Goniaceritae Reitter**
**Tribe Brachyglutini Raffray**
*Brachygluta abdominalis* (Aubé)
*Brachygluta luniger* (LeConte)***
*Decarthron abnorme* (LeConte)*
*Eutrichites zonatus* (Brendel)**
*Reichenbachia borealis* Casey
*Reichenbachia corporalis* Casey**
*Reichenbachia propinqua* (LeConte)
*Reichenbachia spatulifer* Casey
*Rybaxis clavata* (Brendel)
*Rybaxis conjuncta* (LeConte)
*Rybaxis mystica* Casey
*Rybaxis transversa* Fall
*Rybaxis varicornis* (Brendel)
**Tribe Bythinini Raffray**
*Tychobythinus bythinioides* (Brendel)*
**Tribe Tychini Raffray**
*Lucifotychus hirsutus* Chandler*
*Lucifotychus testaceus* (Casey)
**Supertribe Pselaphitae Latreille**
**Tribe Tyrini Reitter**
*Tyrus semiruber* Casey
**Tribe Ctenistini Blanchard**
*Ctenisodes piceus* (LeConte)**
**Tribe Pselaphini Latreille**
*Pselaphus bellax* Casey
*Pselaphus fustifer* Casey

**Notes:** *New to province; **New to Maritime provinces; ***New to Canada.

The classification of the Pselaphinae follows the classification of Chandler (2001).

### Family Staphylinidae Latreille, 1802

**Subfamily Pselaphinae Latreille, 1802**

**Supertribe Euplectitae Streubel, 1839**

**Tribe Euplectini Streubel, 1839**

#### Euplectus acomanus

Casey, 1908**

http://species-id.net/wiki/Euplectus_acomanus

Map 1

##### Material examined.

**New Brunswick, York Co.**, 15 km W of Tracy off Rt. 645, 45.6848°N, 66.8821°W, 20–29.VII.2009, 4–11.VIII.2009, R. Webster & M.-A. Giguère, old red pine forest, Lindgren funnel traps (2 ♂, RWC); same locality and forest type, 4–16.VI.2010, 16–30.VI.2010, R. Webster & C. MacKay, Lindgren funnel traps (2 ♂, RWC); same locality and forest type, 30.VI.–13.VII.2010, R. Webster & K. Burgess (2 ♂, RWC); 14 km WSW of Tracy, S of Rt. 645, 45.6741°N, 66.8661°W, 30.VI-13.VII.2010, R. Webster & K. Burgess, old mixed forest with red and white spruce, red and white pine, balsam fir, eastern white cedar, red maple, and *Populus* sp., Lindgren funnel traps (1 ♂, RWC).

**Map 1.**
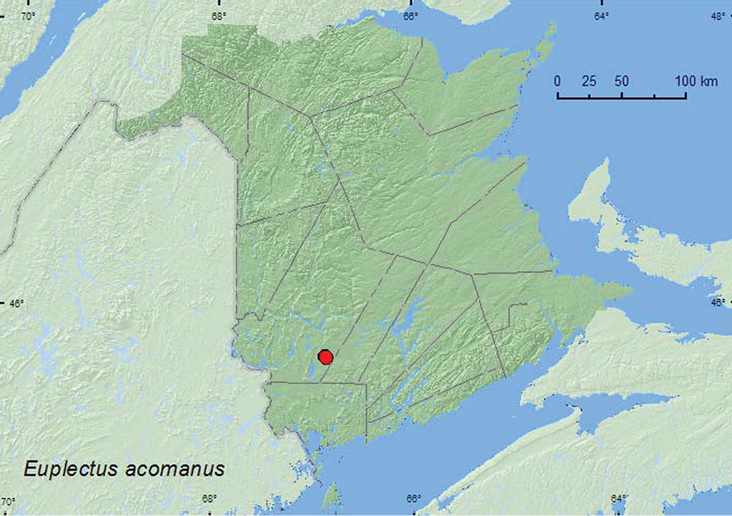
Collection localities in New Brunswick, Canada of *Euplectus acomanus*.

##### Collection and habitat data.

Wagner (1975) recorded this species from under bark of pine (*Pinus* sp.). Chandler (1990) reported that this species was associated with dead pine trees in Latimer Co., Oklahoma, as well as being collected from an oak (*Quercus* sp.) tree hole, old sawdust, under bark, and at the base of a standing dead pine (Chandler 1997). In New Brunswick, adults were captured in Lindgren funnel traps deployed in an old (180-year-old) red pine (*Pinus resinosa* Ait.) forest and an old (180-year-old) mixed forest with various conifer species including red and white pine (*Pinus strobus* L.). Adults were captured during June, July, and August.

##### Distribution in Canada and Alaska.

QC, **NB** (Davies 1991).

#### Euplectus confluens

LeConte, 1849**

http://species-id.net/wiki/Euplectus_confluens

Map 2

##### Material examined.

**New Brunswick, Queens Co.**, Cranberry Lake P.N.A, 46.1125°N, 65.6075°W, 25.VI–1.VII.2009, 10–15.VII.2009, 15–21.VII.2009, 28.VII-6.VIII.2009, R. Webster & M.-A. Giguère, old red oak forest, Lindgren funnel traps (8 ♂, AFC, RWC); Grand Lake Meadows P.N.A., 45.8227°N, 66.1209°W, 31.V-15.VI.2010, R. Webster & C. MacKay, old silver maple forest with green ash and seasonally flooded marsh, Lindgren funnel trap (1 ♂, RWC). **York Co.**, 14 km WSW of Tracy, S of Rt. 645, 45.6741°N, 66.8661°W, 30.VI–13.VII.2010, R. Webster & K. Burgess, old mixed forest with red and white spruce, red and white pine, balsam fir, eastern white cedar, red maple, and *Populus* sp., Lindgren funnel trap (1 ♂, RWC).

**Map 2.**
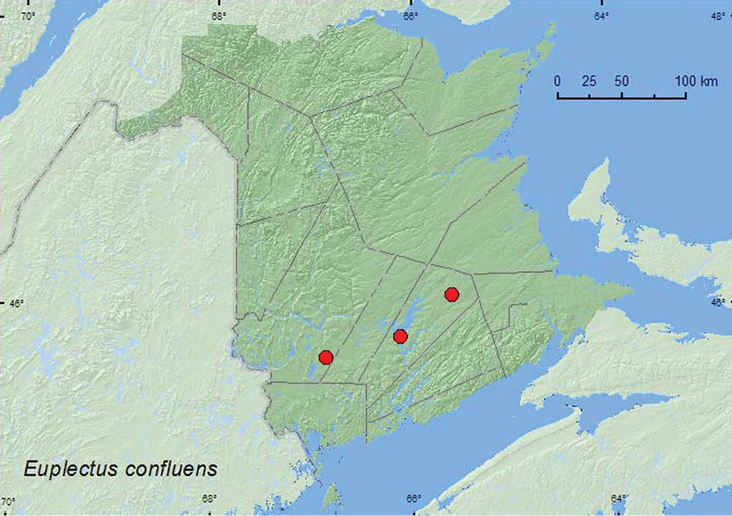
Collection localities in New Brunswick, Canada of *Euplectus confluens*.

##### Collection and habitat data.

Park et al. (1950) reported *Euplectus confluens*from a sugar maple (*Acer saccharum* Marsh.) tree hole. According to Wagner (1975),the preferred habitat of this species was loose, moist, decayed woody debris in hollow trees and basal tree holes in American beech (*Fagus grandifolia* Ehrh.) and sugar maple. Wagner (1975) considered this species to be the most frequently collected *Euplectus* species in eastern deciduous forests. Chandler (1997) reported that this species was most commonly found in tree holes and rotten wood, but was also taken from barn debris and sawdust and at an ultraviolet light. In New Brunswick, adults were captured in Lindgren funnel traps deployed in an old red oak (*Quercus rubra* L.) forest, an old silver maple (*Acer saccharinum* L.) swamp, and an old mixed forest. Basal tree holes were frequent in the red oak and the mixed forest stand. Adults were captured during June, July, and August.

##### Distribution in Canada and Alaska.

QC, **NB** (Davies 1991).

#### Euplectus elongatus

Brendel, 1893

http://species-id.net/wiki/Euplectus_elongatus

Map 3

##### Material examined. 

**New Brunswick, Charlotte Co.**, 10 km NW of New River Beach, 45.2110°N, 66.6170°W, 29.VI-16.VII.2009, R. Webster & C. MacKay, old growth eastern white cedar forest, Lindgren funnel trap (1 ♂, RWC). **York Co.**, 15 km W of Tracy off Rt. 645, 45.6848°N, 66.8821°W, 4–11.V.2009, 11–19.V.2009, R. Webster & M.-A. Giguère, old red pine forest, Lindgren funnel traps (4 ♂, RWC); same locality and forest type, 18.V-2.VI.2010, R. Webster & C. MacKay, Lindgren funnel traps (2 ♂, RWC); same locality and forest type, 27.VII-10.VIII.2010, R. Webster & C. Hughs (1 ♂, RWC).

**Map 3.**
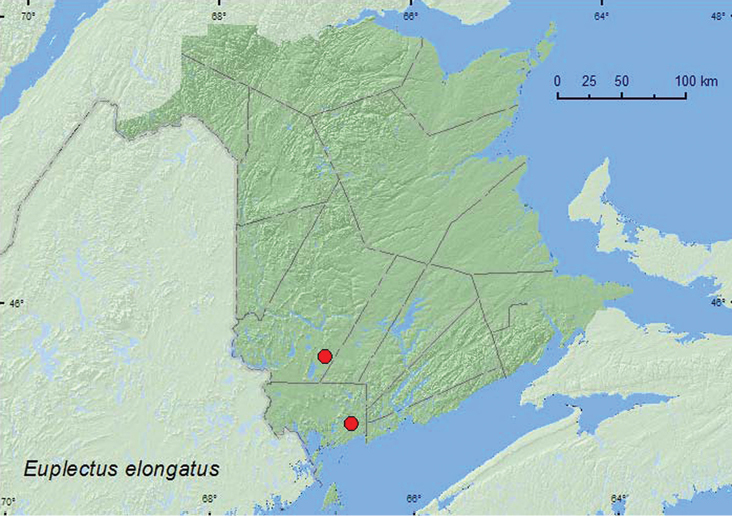
Collection localities in New Brunswick, Canada of *Euplectus elongatus*.

##### Collection and habitat data.

*Euplectus elongatus* has been found in leaf litter and under bark of a decaying log (Wagner 1975). In New Brunswick, adults were captured in Lindgren funnel traps deployed in an old eastern white cedar (*Thuja occidentalis* L.) forest, and an old red pine forest. Adults were collected during June, July, and August.

##### Distribution in Canada and Alaska.

ON, QC, **NB**, NS (Davies 1991; Paquin and Dupérré 2002; Dollin et al. 2008; Bishop et al. 2009).

#### Pycnoplectus linearis

(LeConte, 1849)

http://species-id.net/wiki/Pycnoplectus_linearis

Map 4

##### Material examined.

**New Brunswick, Charlotte Co.**, 10 km NW of New River Beach, 45.2110°N, 66.6170°W, 10–23.VIII.2010, C. Hughes & K. Burgess, old growth eastern white cedar forest, Lindgren funnel trap (1 ♂, RWC). **Queens Co.**, Cranberry Lake P.N.A, 46.1125°N, 65.6075°W, 18–25.VI.2009, 25.VI-1.VII.2009, 1–10.VII.2009, 10–15.VII.2009, R. Webster & M.-A. Giguère, old red oak forest, Lindgren funnel traps (4 ♂, AFC, RWC). **Sunbury Co.**, Acadia Research Forest, 45.9866°N, 66.3841°W, 8–13.V.2009, 21–29.VII.2009, R. Webster & M.-A. Giguère, red spruce forest with red maple and balsam fir, Lindgren funnel traps (2, RWC). **York Co.**, 15 km W of Tracy off Rt. 645, 45.6848°N, 66.8821°W, 7–14.VII.2009, 21–29.VII.2009, R. Webster & M.-A. Giguère, old red pine forest, Lindgren funnel traps (2 ♂, RWC); same locality and forest type, 10–26.V.2010, 4–16.VI.2010, R. Webster & C. MacKay, Lindgren funnel traps (2 ♂, RWC).

**Map 4.**
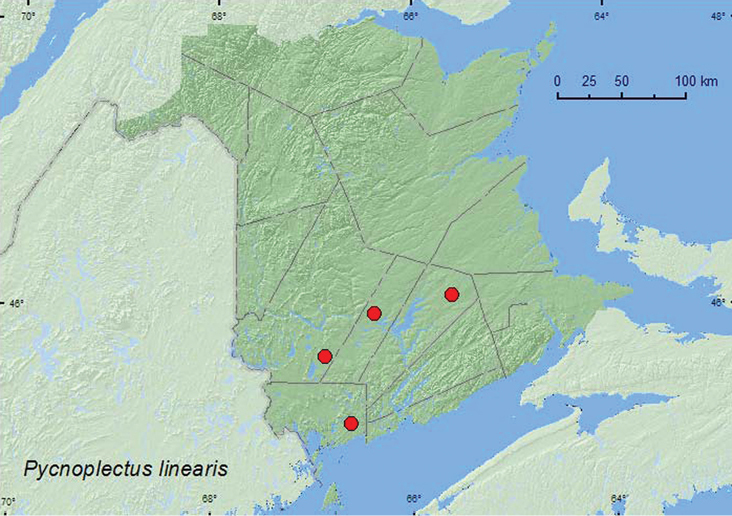
Collection localities in New Brunswick, Canada of *Pycnoplectus linearis*.

##### Collection and habitat data.

This species has been collected from log mold, sawdust, tree holes, and under bark (Wagner 1975). The adults from New Brunswick were collected from Lindgren funnel traps deployed in an old eastern white cedar forest, an old red oak forest, a 110-year-old red spruce (*Picea rubens* Sarg.) forest, and an old red pine forest. Adults were captured during May, June, July, and August.

##### Distribution in Canada and Alaska.

ON, **NB**, NS (Davies 1991; Dollin et al. 2008).

### Tribe Trichonychini Reitter, 1882

#### Actiastes foveicollis

(LeConte, 1878)**

http://species-id.net/wiki/Actiastes_foveicollis

Map 5

##### Material examined. 

**New Brunswick, Queens Co.**, Cranberry Lake P.N.A, 46.1125°N, 65.6075°W, 11–18.VI.2009, 28.VII-6.VIII.2009, 6–14.VIII.2009, R. Webster & M.-A. Giguère, old red oak forest, Lindgren funnel traps (3, RWC); Grand Lake Meadows P.N.A., 45.8227°N, 66.1209°W, 12–26.VII.2010, R. Webster & C. MacKay, old silver maple forest with green ash and seasonally flooded marsh, Lindgren funnel trap (1, RWC). **York Co.**, 15 km W of Tracy off Rt. 645, 45.6848°N, 66.8821°W, 15–21.VI.2009, 7–14.VII.2009, 11–18.VIII.2009, R. Webster & M.-A. Giguère, old red pine forest, Lindgren funnel traps (4, AFC, RWC); same locality and forest type, 16–30.VI.2010, R. Webster & C. MacKay, Lindgren funnel traps (2, RWC); 14 km WSW of Tracy, S of Rt. 645, 45.6741°N, 66.8661°W, 26.IV–10.V.2010, 16–30.VI.2010, 30.VI–13.VII.2009, R. Webster & C. MacKay, old mixed forest with red and white spruce, red and white pine, balsam fir, eastern white cedar, red maple, and *Populus* sp., Lindgren funnel traps (4, AFC, RWC).

**Map 5.**
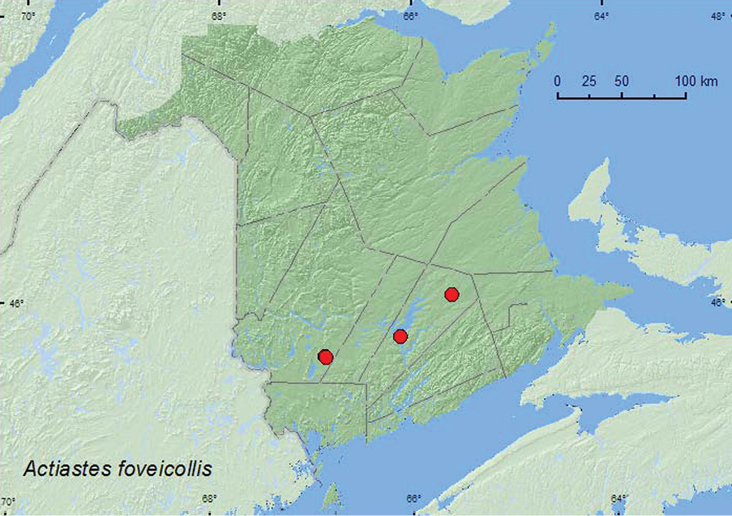
Collection localities in New Brunswick, Canada of *Actiastes foveicollis*.

##### Collection and habitat data.

This species was collected from leaf litter in New Hampshire (Chandler 1987), and is associated with hardwood leaf litters, often near water, and has been taken infrequently from rotten wood (Chandler 1997).In New Brunswick, this species was captured in Lindgren funnel traps deployed in an old red oak forest, an old silver maple forest, an old red pine forest, and an old mixed forest. Adults were captured during April, May, June, July, and August.

##### Distribution in Canada and Alaska.

BC, ON, QC, **NB** (Grigarick and Schuster 1971; Davies 1991).

#### Actiastes globiferum

(LeConte, 1849)**

http://species-id.net/wiki/Actiastes_globiferum

Map 6

##### Material examined. 

**New Brunswick, Charlotte Co.**, 10 km NW of New River Beach, 45.2110°N, 66.6170°W, 29.VI-16.VII.2009, 16–26.VII.2010, R. Webster & C. MacKay, old growth eastern white cedar forest, Lindgren funnel traps (2, AFC). **Queens Co.**, Cranberry Lake P.N.A, 46.1125°N, 65.6075°W, 27.V–5.VI.2009, 11–18.VI.2009, 25.VI-1.VII.2009, 1–10.VII.2009, 10–15.VII.2009, 15–21.VII.2009, R. Webster & M.-A. Giguère, old red oak forest, Lindgren funnel traps (10, AFC, NBM, RWC). **Restigouche Co.**, Dionne Brook P.N.A., 47.9030°N, 68.3503°W, 27.VI–14.VII.2011, 28.VII-9.VIII.2011, M. Roy & V. Webster, old-growth northern hardwood forest, Lindgren funnel traps (2, AFC, NBM); same locality and collectors but 47.9064°N, 68.3441°W, 27.VI-14.VII.2011, 14–28.VII.2011, 28.VII–9.VIII.2011, old-growth white spruce and balsam fir forest, Lindgren funnel traps (5, NBM, RWC). **Sunbury Co.**, Acadia Research Forest, 45.9866°N, 66.3841°W, 13–21.VII.2009, R. Webster & M.-A. Giguère, red spruce forest with red maple and balsam fir, Lindgren funnel trap (1, AFC). **York Co.**, 15 km W of Tracy off Rt. 645, 45.6848°N, 66.8821°W, 19–25.V.2009, 1–8.VI.2009, 8–15.VI.2009, 15–21.VI.2009, 21–28.VI.2009, 28.VI–7.VII.2009, 20–29.VII.2009, R. Webster & M.-A. Giguère, old red pine forest, Lindgren funnel traps (9, AFC, RWC).

**Map 6.**
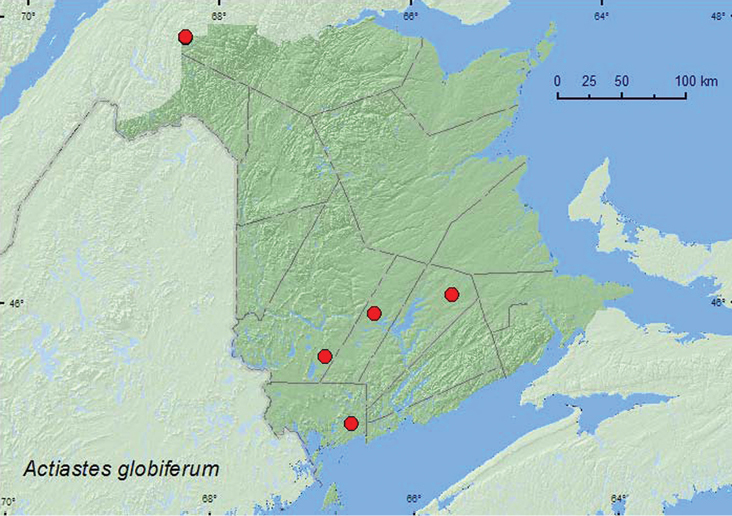
Collection localities in New Brunswick, Canada of *Actiastes globiferum*.

##### Collection and habitat data.

Chandler (1997) notes that specimens have been taken from under pine bark, from pine and oak litter, and from tree holes. In New Brunswick, *Actiastes globiferum* was captured in Lindgren funnel traps deployed in an old-growth eastern white cedar forest, a 110-year-old red spruce forest, an old red pine forest, and an old red oak forest. Adults were collected during May, June, July, and August.

##### Distribution in Canada and Alaska.

QC, **NB** (Davies 1991).

#### Bibloporus bicanalis

(Casey, 1884)

http://species-id.net/wiki/Bibloporus_bicanalis

Map 7

##### Material examined.

**New Brunswick, Carleton Co.**, Jackson Falls, Bell Forest, 46.2200°N, 67.7231°W, 4–12.VI.2008, 19–27.VI.2008, 27.VI-5.VII.2008, 5–12.VII.2008, R. P. Webster, mature hardwood forest, Lindgren funnel traps (6, AFC, NBM, RWC); same locality and forest type, 23–28.IV.2009, 28.IV–9.V.2009, 9–14.V.2009, 14–20.V.2009, 20–26.V.2009, 26.V–1.VI.2009, 1–8.VI.2009, R. Webster & M.-A. Giguère, Lindgren funnel traps (16, AFC, RWC). **Charlotte Co.**, 10 km NW of New River Beach, 45.2110°N, 66.6170°W, 17–31.V.2010, R. Webster & C. MacKay, old growth eastern white cedar forest, Lindgren funnel trap (1, AFC). **Queens Co.**, Cranberry Lake P.N.A (Protected Natural Area), 46.1125°N, 65.6075°W, 24.IV–5.V.2009, 5–12.V.2009, 12–21.V.2009, 21–27.V.2009, 5–11.VI.2009, 11–18.VI.2009, R. Webster & M.-A. Giguère, old red oak forest, Lindgren funnel traps (9, AFC, RWC). **Restigouche Co.**, Dionne Brook P.N.A., 47.9030°N, 68.3503°W, 30.V–15.VI.2011, M. Roy & V. Webster, old-growth northern hardwood forest, Lindgren funnel traps (2, AFC, NBM); same locality and collectors but 47.9064°N, 68.3441°W, 31.V–15.VI.2011, old-growth white spruce and balsam fir forest, Lindgren funnel trap (1, NBM). **Sunbury Co.**, Acadia Research Forest, 45.9866°N, 66.3841°W, 2–9.VI.2009, 16–24.VI.2009, 24–30.VI.2009, R. Webster & M.-A. Giguère, red spruce forest with red maple and balsam fir (110 years-old), Lindgren funnel traps (5, AFC). **York Co.**, 15 km W of Tracy off Rt. 645, 45.6848°N, 66.8821°W, 25.IV–4.V.2009, 4–11.V.2009, 11–19.V.2009, 1–8.VI.2009, 8–15.VI.2009, 15–21.VI.2009, R. Webster & M.-A. Giguère, old red pine forest, Lindgren funnel traps (8, AFC, RWC); 14 km WSW of Tracy, S of Rt. 645, 45.6741°N, 66.8661°W, 26.IV–10.V.2010, 10–26.V.2010, R. Webster & C. MacKay, old mixed forest with red and white spruce, red and white pine, balsam fir, eastern white cedar, red maple, and *Populus* sp., Lindgren funnel traps (4, AFC, RWC); Charters Settlement, 45.8288°N, 66.7365°W, 1–11.IX.2008, R. P. Webster, mature mixed forest, Lindgren funnel trap (1, RWC).

**Map 7.**
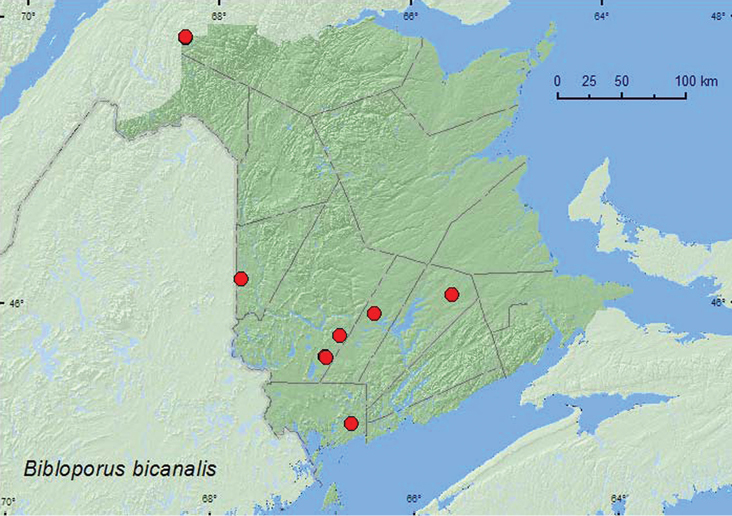
Collection localities in New Brunswick, Canada of *Bibloporus bicanalis*.

##### Collection and habitat data.

*Bibloporus bicanalis* was reported from white pine litter (Chandler 1997), and Klimaszewski et al. (2007) indicated that the largest numbers have been taken using intercept traps in old-growth forests, particularly where old conifers are present. In New Brunswick, adults were captured Lindgren funnel traps deployed in a mature hardwood forest with sugar maple, American beech, and white ash (*Fraxinus americana* L.), an old red oak forest, an old mixed forest, an old-growth northern hardwood forest with sugar maple and yellow birch, a mature mixed forest, an old red pine forest, an old eastern white cedar forest/swamp, a 110-year-old red spruce forest, and an old-growth white spruce and balsam fir forest (boreal forest). This species was most frequently captured in hardwood forests. Adults were captured during April, May, June, and September.

##### Distribution in Canada and Alaska.

QC, **NB,** NS(Chandler 1997; Dollin et al. 2008; Bishop et al. 2009).

#### Dalmosella tenuis

Casey, 1897***

http://species-id.net/wiki/Dalmosella_tenuis

Map 8

##### Material examined.

**CANADA, New Brunswick, Charlotte Co.**, 10 km NW of New River Beach, 45.2110°N, 66.6170°W, 29.VI-16.VII.2010, R. Webster & C. MacKay, old eastern white cedar forest, Lindgren funnel trap (1, AFC). **Queens Co.**, Cranberry Lake P.N.A, 46.1125°N, 65.6075°W, 18–25.VI.2009, 14–19.VIII.2009, R. Webster & M.-A. Giguère, old red oak forest, Lindgren funnel traps (2, RWC): same locality data and forest type, 7–13.VII.2011, M. Roy & V. Webster, Lindgren funnel trap (1, RWC). **York Co.**, 15 km W of Tracy off Rt. 645, 45.6848°N, 66.8821°W, 21–28.VI.2009, 4–11.VIII.2009, R. Webster & M.-A. Giguère, old red pine forest, Lindgren funnel traps (2, RWC); same locality and forest type, 30.VI-13.VII.2010, R. Webster & K. Burgess, Lindgren funnel trap (1, RWC).

**Map 8.**
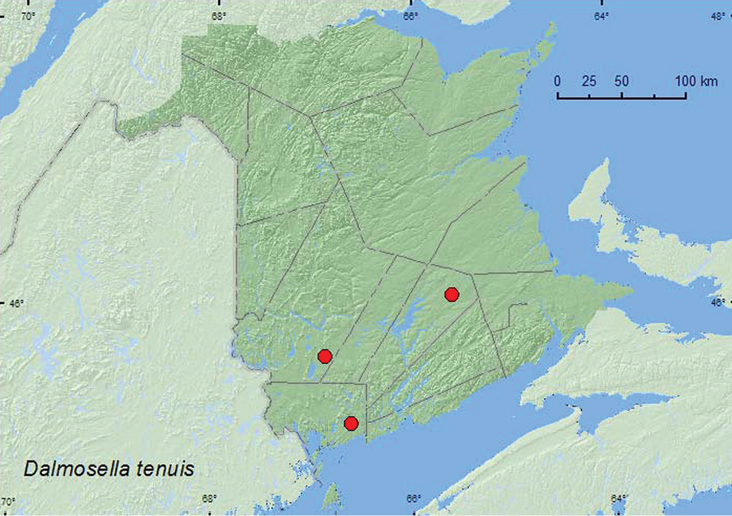
Collection localities in New Brunswick, Canada of *Dalmosella tenuis*.

##### Collection and habitat data. 

Chandler (1990) reported that this species had been taken from various rotten woods and from tree holes. Specimens from New Brunswick were captured in Lindgren funnel traps deployed in an old-growth eastern white cedar forest, old red oak forest, and an old red pine forest. Adults were captured during June and July.

##### Distribution in Canada and Alaska.

**NB** (new Canadian record). Chandler (1997) reported this species from Louisiana east to Florida and north to Maine in the United States.

#### Ramecia crinita

(Brendel, 1865)

http://species-id.net/wiki/Ramecia_crinita

Map 9

##### Material examined.

**New Brunswick, Queens Co.**, Cranberry Lake P.N.A, 46.1125°N, 65.6075°W, 21–27.V.2009, 5–11.VI.2009, 18–25.VI.2009, 25.VI–1.VII.2009, R. Webster & M.-A. Giguère, old red oak forest, Lindgren funnel traps (4, AFC, RWC); same locality data and forest type, 13–25.V.2011, 25.V–7.VI.2011, 7–22.VI.2011, M. Roy & V. Webster, Lindgren funnel traps (4, NBM, RWC). **York Co.**, 15 km W of Tracy off Rt. 645, 45.6848°N, 66.8821°W, 15–21.VI.2009, R. Webster & M.-A. Giguère, old red pine forest, Lindgren funnel traps (5, RWC); same locality and forest type, 4–16.VI.2010, 16–30.VI.2010, R. Webster & C. MacKay, Lindgren funnel traps (2, RWC).

**Map 9.**
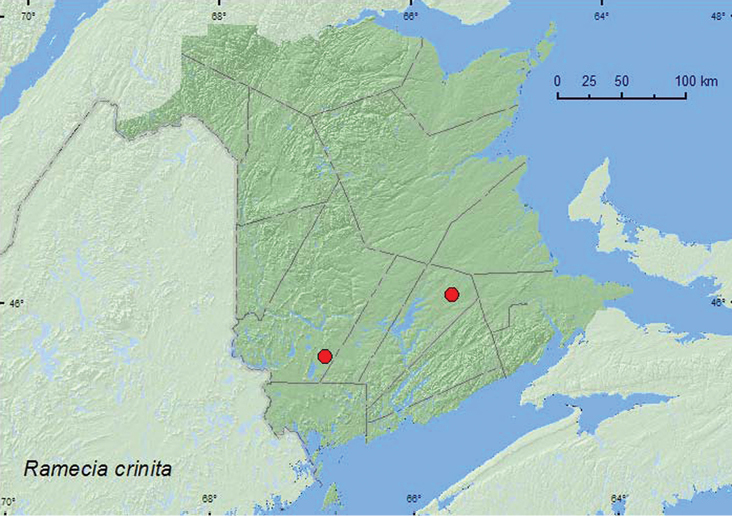
Collection localities in New Brunswick, Canada of *Ramecia crinita*.

##### Collection and habitat data.

This species has been found under bark of maple and oak (Chandler 1997). Specimens from New Brunswick were captured in Lindgren funnel traps deployed in an old red oak forest, and an old red pine forest. Adults were captured during May and June.

##### Distribution in Canada and Alaska.

QC, **NB**, NS (Davies 1991).

#### Trimioplectus obsoletus

Brendel, 1891**

http://species-id.net/wiki/Trimioplectus_obsoletus

Map 10

##### Material examined.

**New Brunswick, Queens Co.**, Cranberry Lake P.N.A, 46.1125°N, 65.6075°W, 11–18.VI.2009, 18–25.VI.2009, 25.VI-1.VII.2009, 15–21.VII.2009, 21–28.VII.2009, R. Webster & M.-A. Giguère, old red oak forest, Lindgren funnel traps (9, AFC, RWC). **Sunbury Co.**, Acadia Research Forest, 45.9866°N, 66.3841°W, 24–30.VI.2009, 30.VI-8.VII.2009, 8–13.VII.2009, 13–21.VII.2009, R. Webster & M.-A. Giguère, red spruce forest with red maple and balsam fir, Lindgren funnel traps (8, AFC, RWC). **York Co.**, 15 km W of Tracy off Rt. 645, 45.6848°N, 66.8821°W, 15–21.VI.2009, 20–29.VII.2009, R. Webster & M.-A. Giguère, old red pine forest, Lindgren funnel traps (3, AFC, RWC); 14 km WSW of Tracy, S of Rt. 645, 45.6741°N, 66.8661°W, 10–26.V.2010, R. Webster & C. MacKay, old mixed forest with red and white spruce, red and white pine, balsam fir, eastern white cedar, red maple, and *Populus* sp., Lindgren funnel traps (2, AFC).

**Map 10.**
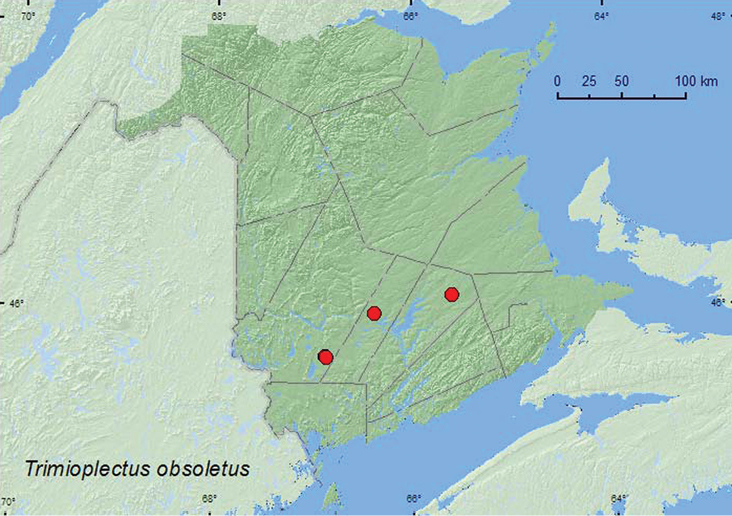
Collection localities in New Brunswick, Canada of *Trimioplectus obsoletus*.

##### Collection and habitat data.

Park (1949), Park et al. (1950), and Chandler (1990) reported that this species was most commonly collected from hardwood tree holes and rotten wood. Specimens from New Brunswick were captured in Lindgren funnel traps deployed in an 110-year-old red spruce forest with red maple (*Acer rubrum* L.), an old red pine forest, an old mixed forest, and an old red oak forest. Adults were captured during May, June, and July.

##### Distribution in Canada and Alaska.

ON, QC, **NB** (Davies 1991).

### Supertribe Batrisitae Reitter, 1882

**Tribe Batrisini Reitter, 1882**

#### Batrisodes frontalis

(LeConte, 1849)**

http://species-id.net/wiki/Batrisodes_frontalis

Map 11

##### Material examined.

**New Brunswick, Carleton Co.**, Jackson Falls, Bell Forest, 46.2200°N, 67.7231°W, 5–12.VII.2008, R. P. Webster, mature hardwood forest, Lindgren funnel trap (1, RWC). **Queens Co.**, Cranberry Lake P.N.A, 46.1125°N, 65.6075°W, 31.V–11.VI.2009, 11–18.VI.2009, 25.VI-1.VII.2009, 15–21.VII.2009, 28.VII-6.VIII.2009, R. Webster & M.-A. Giguère, old red oak forest, Lindgren funnel traps (6, AFC, NBM, RWC); Grand Lake Meadows P.N.A., 45.8227°N, 66.1209°W, 12–26.VII.2010, R. Webster & C. MacKay, old silver maple forest with green ash and seasonally flooded marsh, Lindgren funnel trap (1, RWC). **Sunbury Co.**, Acadia Research Forest, 45.9866°N, 66.3841°W, 13–21.VII.2009, 21–29.VII.2009, R. Webster & M.-A. Giguère, red spruce forest with red maple and balsam fir, Lindgren funnel traps (2, RWC). **York Co.**, 15 km W of Tracy off Rt. 645, 45.6848°N, 66.8821°W, 17–26.VII.2008, R. P. Webster, old red pine forest, Lindgren funnel trap (1, AFC); same locality and forest type but 21–28.VI.2009, 28.VI–7.VII.2009, R. Webster & M.-A. Giguère, Lindgren funnel traps (3, RWC).

**Map 11.**
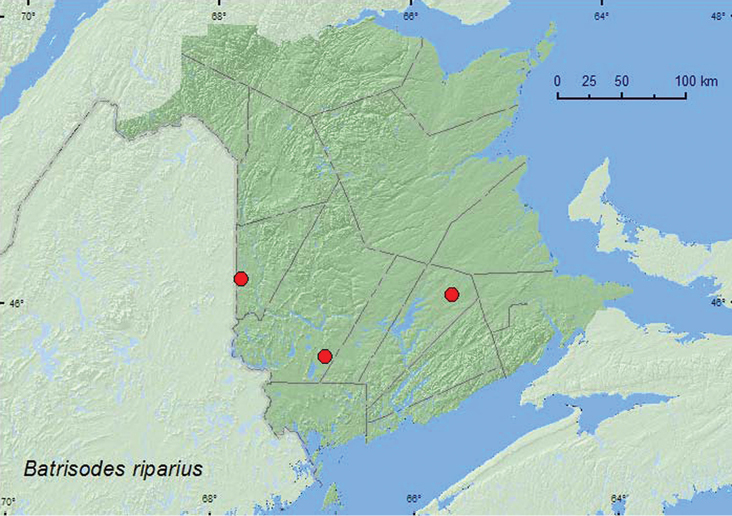
Collection localities in New Brunswick, Canada of *Batrisodes frontalis*.

##### Collection and habitat data.

*Batrisodes*speciesare usually found in leaf litter, rotten wood on the forest floor, or in ant nests amongst the ants, particularly beneath the bark of conifers (Chandler 2000). They are known to be predators or scavengers on mites, earthworms, and the brood of ants (Park 1947). *Batrisodes frontalis* has been reported from beneath bark in the nests of three species of *Lasius* ants (Wickham 1898, 1900; Park 1947). All specimens from New Brunswick were captured in Lindgren funnel traps deployed in a mature hardwood forest, an old red oak forest, an old silver maple forest, a 110-year-old red spruce forest, and an old red pine forest. Adults were captured during June, July, and August.

##### Distribution in Canada and Alaska.

AB, MB**,** ON, QC, **NB** (Davies 1991).

#### Batrisodes riparius

(Say, 1824)**

http://species-id.net/wiki/Batrisodes_riparius

Map 12

##### Material examined.

**New Brunswick, Carleton Co.**, Jackson Falls, Bell Forest, 46.2200°N, 67.7231°W, 5–12.VII.2008, R. P. Webster, mature hardwood forest, Lindgren funnel trap (1, RWC). **Queens Co.**, Cranberry Lake P.N.A, 46.1125°N, 65.6075°W, 10–15.VII.2009, R. Webster & M.-A. Giguère, old red oak forest, Lindgren funnel trap (1, RWC). **York Co.**, 15 km W of Tracy off Rt. 645, 45.6848°N, 66.8821°W, 21–27.VIII.2008, 21–28.VI.2009, 14–20.VII.2009, 20–29.VII.2009, R. Webster & M.-A. Giguère, old red pine forest, Lindgren funnel traps (5, AFC, RWC); same locality data and forest type, 30.VI-13.VII.2010, R. Webster & K. Burgess, Lindgren funnel trap (1, RWC).

**Map 12.**
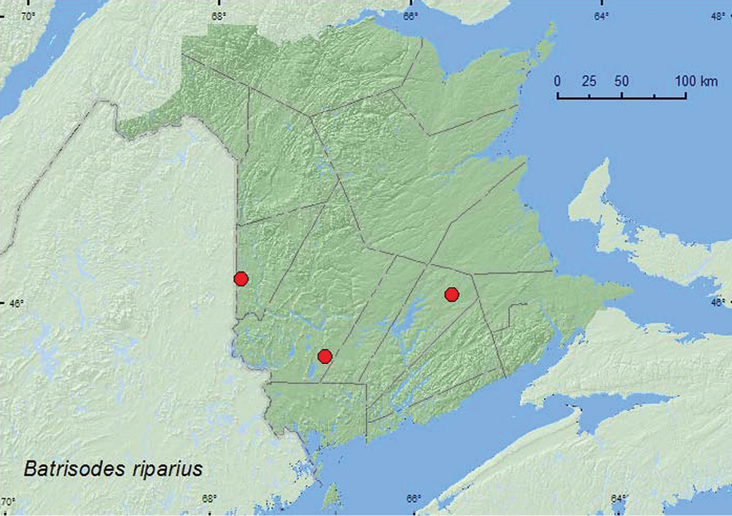
Collection localities in New Brunswick, Canada of *Batrisodes riparius*.

##### Collection and habitat data.

*Batrisodes riparius*was reported from an ant (*Aphaenogaster*) nest by Park (1947), and has been taken beneath bark, from tree holes, and from rotten wood by fallen trees (Chandler 1997). Specimens from New Brunswick were captured in Lindgren funnel traps deployed in a mature hardwood forest, an old red oak forest, and an old red pine forest. Adults were collected during June, July, and August.

##### Distribution in Canada and Alaska.

ON, QC, **NB** (Davies 1991).

#### Batrisodes scabriceps

(LeConte, 1849)**

http://species-id.net/wiki/Batrisodes_scabriceps

Map 13

##### Material examined.

**New Brunswick, Queens Co.**, Cranberry Lake P.N.A, 46.1125°N, 65.6075°W, 11–18.VI.2009, 18–25.VI.2009, 1–10.VII.2009, 10–15.VII.2009, 28.VII–6.VIII.2009, R. Webster & M.-A. Giguère, old red oak forest, Lindgren funnel traps (8, AFC, RWC).

**Map 13.**
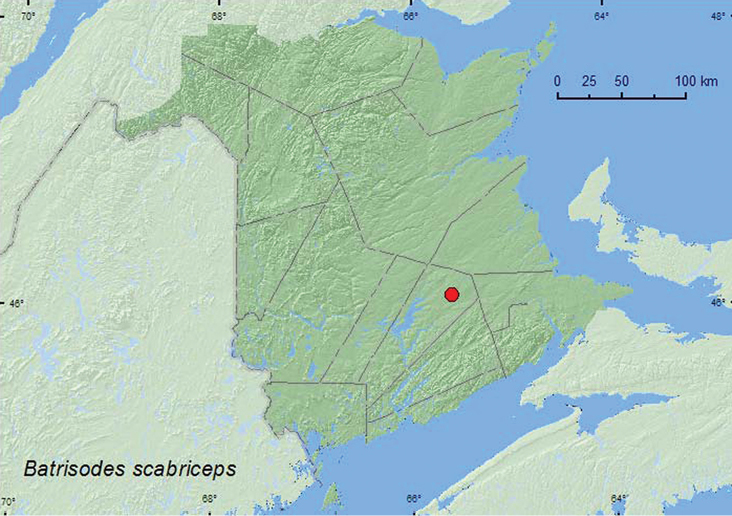
Collection localities in New Brunswick, Canada of *Batrisodes scabriceps*.

##### Collection and habitat data.

*Batrisodes scabriceps*was reported from nests of *Lasius*, *Formica*, and *Aphaenogaster* species of ants (Park 1947). This species has also been taken from beneath oak bark (Chandler 1997), and specimens have been frequently taken in the United States by use of intercept traps or Lindgren funnel traps for bark beetle surveys in pine forests (Klimaszewski et al. 2007). All New Brunswick specimens were captured in Lindgren funnel traps deployed in an old red oak forest. Adults were captured during June, July, and August.

##### Distribution in Canada and Alaska.

ON, QC, **NB** (Davies 1991; Klimaszewski et al. 2007).

### Supertribe Goniaceritae Reitter, 1882

**Tribe Brachyglutini Raffray, 1904**

#### Brachygluta luniger

(LeConte, 1849)***

http://species-id.net/wiki/Brachygluta_luniger

Map 14

##### Material examined.

**New Brunswick, Gloucester Co.**, near Acadian Historical Village, 47.7873°N, 65.0797°W, 14.VIII.2005, R. P. Webster & G. Pohl, salt marsh, intertidal zone, on patches of bare clay at base of *Spartina patens* on upper margin of tidal stream (10, RWC).

**Map 14.**
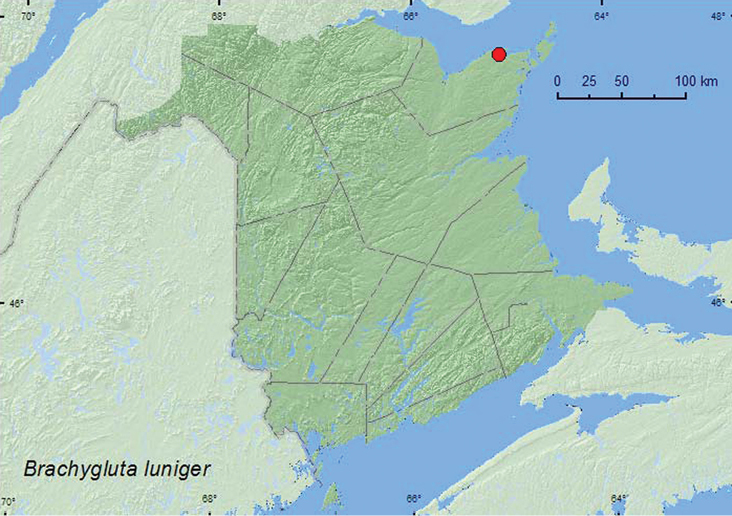
Collection localities in New Brunswick, Canada of *Brachygluta luniger*.

##### Collection and habitat data.

*Brachygluta luniger* was collected in the intertidal zone of a salt marsh and is known to be associated with salt marshes (Chandler 1997). The New Brunswick adults were found on small patches of bare clay at the base of salt-meadow grass, *Spartina patens* (Ait.) Muhl., on the upper margin of a tidal stream. Adults were collected during August.

##### Distribution in Canada and Alaska.

**NB** (new Canadian record). This species has been reported from Massachusetts south to Florida in the United States (Chandler 1997).

#### Decarthron abnorme

(LeConte, 1849)

http://species-id.net/wiki/Decarthron_abnorme

Map 15

##### Material examined.

**New Brunswick, Albert Co.**, Shepody N.W.A., New Horton Section, 45.6940°N, 64.7000°W, 29.VI.2004, R. P. Webster, cattail marsh, treading (1, RWC). **Carleton Co.**, Two Mile Brook Fen, 46.3619°N, 67.6733°W, 6.V.2005, M.-A. Giguère & R. P. Webster, old growth eastern white cedar swamp, in litter at base of cedar (1, RWC);trail to Two Mile Brook Fen, 46.3510°N, 67.6815°W, 6.V.2005, M.-A. Giguère & R. P. Webster, cattail and *Carex* marsh, in leaf litter on marsh margin (1, RWC). **Charlotte Co.**, S of Little Pocologan River, 45.1537°N, 66.2669°W, 7.V.2007, R. P. Webster, black spruce and tamarack bog, in litter and moss (1, NBM). **Madawaska Co.**, Loon Lake, 236 m elev., 47.7839°N, 68.3943°W, 21.VII.2010, R. P. Webster, boreal forest, small lake surrounded by sedges, treading sedges near *Myrica* bushes (1 ♂, NBM). **Saint John Co.**, Chance Harbour off Rt. 790, 45.1374°N, 66.3633°W, 15.V.2006, R. P. Webster, raised peatland (with black spruce), treading saturated sphagnum (1, RWC). **Sunbury Co.**, Acadia Research Forest, 46.0173°N, 66.3741°W, 18.VI.2007, R. P. Webster, 8.5 year-old regenerating mixed forest, in sphagnum and leaf litter at bottom of old tire depression (1, RWC). **York Co.**, Charters Settlement, 45.8282°N, 66.7367°W, 9.IV.2005, 29.III.2006, R. P. Webster, *Carex* marsh, in leaf litter at base of trees and shrubs (4, NBM, RWC); same locality and collector but 45.8430°N, 66.7280°W, 29.IX.2004, small sedge marsh, in moist litter (1, RWC): same locality and collector but 45.8395°N, 66.7391°W, 17.VII.2004, 27.VI.2006, 25.VI.2009, mixed forest, u.v. light (6, NBM, RWC); Canterbury, Browns Mountain Fen, 45.8967°N, 67.6343°W, 2.V.2005, M.-A. Giguère & R. P. Webster, calcareous cedar fen, in moss and litter at base of tree (cedar) (1, RWC); 8.4 km W of Tracy off Rt. 645, 45.68217°N, 66.7894°W, 14.V.2008, R. P. Webster, wet alder swamp, in leaf and grass litter on hummocks (1, NBM).

**Map 15.**
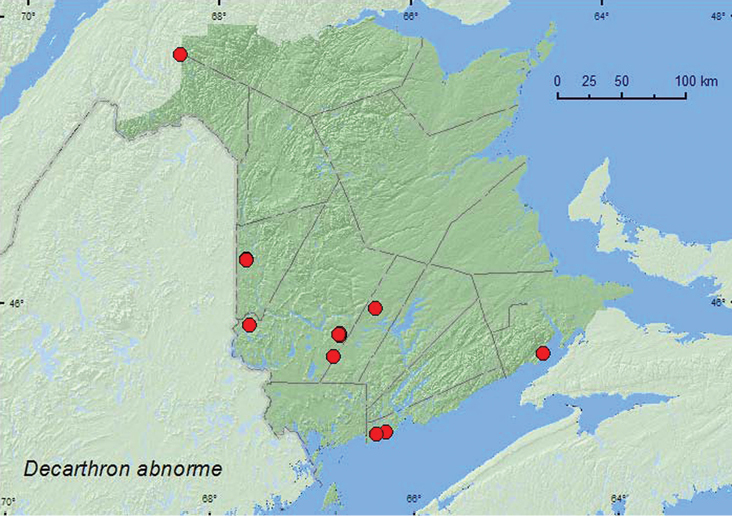
Collection localities in New Brunswick, Canada of *Decarthron abnorme*.

##### Collection and habitat data.

In New Brunswick, this common species was collected in various wetland habitats. These included *Carex* marshes, *Carex* marsh with scattered cattails (*Typha* sp.), a cattail marsh, a black spruce (*Picea mariana* (Mill.) B.S.P.) and tamarack (*Larix laricina* (Du Roi) K. Koch) bog, a coastal raised peatland with black spruce, an old eastern white cedar swamp, a regenerating mixed forest, along a lake margin among *Carex*, and in a wet alder (*Alnus* sp.) swamp. Adults were sifted from moss and litter at bases of trees and marsh margins, sphagnum and leaf litter, and leaf and grass litter on hummocks (alder swamp). Other adults were collected by treading vegetation in cattail and *Carex* marshes, *Carex* near *Myrica* bushes on a lake margin, and a saturated sphagnum mat in bog. Some adults were collected at an ultraviolet light near a mixed forest. This species is most commonly taken from leaf litter along the margins of streams, ponds, and marshes, and from sphagnum moss (Chandler 1997). Adults were captured during late March, April, May, June, July, and September.

##### Distribution in Canada and Alaska.

NT, BC, AB, SK, MB, ON, QC, **NB**, NS (Davies 1991; Majka et al. 2011; CNC specimens) [reported from NB by Majka et al. (2011) in error, C. Majka, personal communication].

#### Eutrichites zonatus

(Brendel, 1865)**

http://species-id.net/wiki/Eutrichites_zonatus

Map 16

##### Material examined.

**New Brunswick, York Co.**, Fredericton, at Saint John River, 45.9588°N, 66.6254°W, 4.VII.2004, R. P. Webster, river margin, in drift material (mostly maple seeds) (1, RWC); Charters Settlement, 45.8395°N, 66.7391°W, 27.VI.2006, 25.VI.2009, R. P. Webster, mixed forest, u.v. light (5, RWC).

**Map 16.**
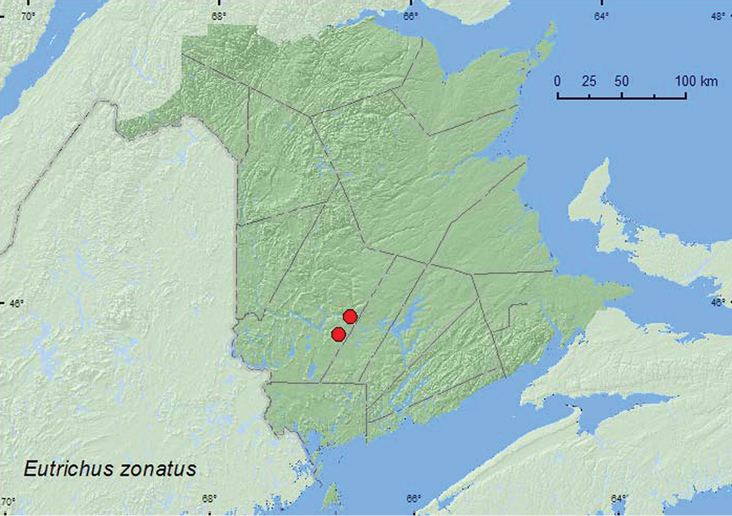
Collection localities in New Brunswick, Canada of *Eutrichites zonatus*.

##### Collection and habitat data.

One specimen was sifted from drift material consisting mostly of maple seeds along a river margin. Other adults were captured at an ultraviolet light deployed near a mixed forest. Members of this species have been taken from grass debris, old river drift, and in sawdust and can be commonly taken at lights in the United States (Chandler 1997). Adults were captured during June and July.

##### Distribution in Canada and Alaska.

ON, QC, **NB** (Davies 1991).

#### Reichenbachia corporalis

Casey, 1897**

http://species-id.net/wiki/Reichenbachia_corporalis

Map 17

##### Material examined. 

**New Brunswick, Carleton Co.**, Two Mile Brook Fen, 46.3619°N, 67.6733°W, 6.V.2005, M.-A. Giguère & R. P. Webster, old growth eastern white cedar swamp, in litter at base of cedar (1, RWC). **Charlotte Co.**, *ca*. 9 km NW of New River, 45.2117°N, 66.6436°W, 13.VI.2008, R. P. Webster, eastern white cedar swamp, in sphagnum and grasses under alders (1, NBM). **Northumberland Co.**, Goodfellow Brook P.N.A., 46.8943°N, 65.3796°W, 23.V.2007, R. P. Webster, old growth eastern white cedar swamp (many vernal pools), in litter, grasses and moss on hummocks near water (1, RWC). **Queens Co.**, W of Jemseg at “Trout Creek”, 45.8227°N, 66.1240°W, 9.V.2004, 4.VI.2004, R. P. Webster, silver maple swamp, sifting litter at base of large tree (2, RWC). **Sunbury Co.**, Portobello Creek N.W.A., Maugerville, 45.8992°N, 66.4248°W, 18.VI.2004, R. P. Webster, silver maple forest, u.v. light trap (1, RWC); Sunpoke Lake marsh, 45.7663°N, 66.5537°W, 11.IX.2005, R. P. Webster, seasonally flooded marsh, in moist litter under *Myrica gale* L. bushes (1, RWC); Acadia Research Forest, 45.9866°N, 66.3841°W, 2–9.VI.2009, R. Webster & M.-A. Giguère, red spruce forest with red maple and balsam fir, Lindgren funnel trap (1, AFC). **York Co.**, Charters Settlement, 45.8282°N, 66.7367°W, 9.IV.2005, R. P. Webster, *Carex* marsh, in leaf litter at base of trees and shrubs (2, RWC); same locality and collector but 45.8428°N, 66.7235°W, 9.IX.2005, 1.IV.2006, mixed forest, in leaf litter and moss near small brook (2, RWC); Mazerolle Settlement, 45.8729°N, 66.8311°W, 9.IV.2006, R. P. Webster, stream margin, in leaf litter at base of northern (eastern) white cedar (3, NBM).

**Map 17.**
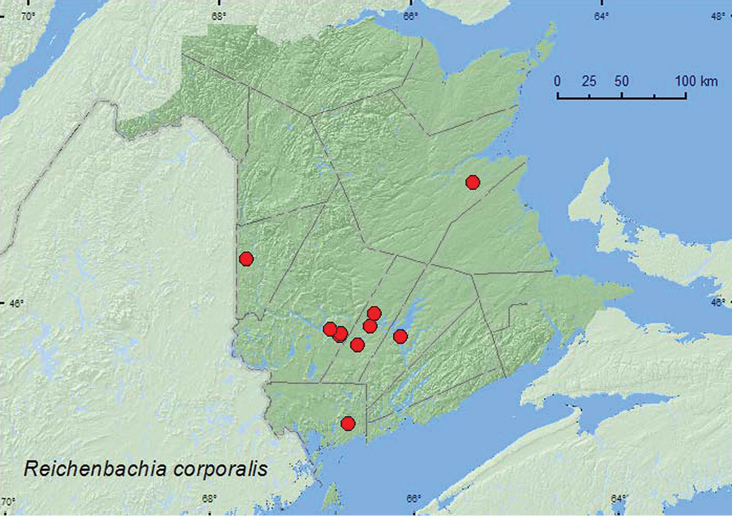
Collection localities in New Brunswick, Canada of *Reichenbachia corporalis*.

##### Collection and habitat data.

In New Brunswick, adults of this species were collected in old eastern white cedar swamps and forests, silver maple swamps, a red spruce forest, a mixed forest, a *Carex* marsh, and a seasonally flooded marsh. Adults were sifted from leaf and/or conifer litter at the bases of trees, from sphagnum and grasses under alders and on hummocks near vernal pond margins, in moist leaf litter under *Myrica gale* L. bushes, and from leaf litter and moss near brook margins. One adult was collected in an ultraviolet light trap and another from a Lindgren funnel trap. Members of this species have been taken from sphagnum moss and from leaf litter of an alder/birch (*Betula* sp.) mixture at the edge of a freshwater marsh (Chandler 1997). Adults were captured during April, May, June, and September.

##### Distribution in Canada and Alaska.

MB,ON, QC, **NB** (Davies 1991; Carlton 2003) [reported from NB by Majka et al. (2011) in error, C. Majka, personal communication].

### Tribe Bythinini Raffray, 1890

#### Tychobythinus bythinioides

(Brendel, 1865)

http://species-id.net/wiki/Tychobythinus_bythinioides

Map 18

##### Material examined.

**New Brunswick, Queens Co.**, Upper Gagetown, bog adjacent to Hwy 2, 45.8316°N, 66.2346°W, 3.IV.2006, R. P. Webster, tamarack bog, in sphagnum hummocks on bog margin (1, RWC); Cranberry Lake P.N.A, 46.1125°N, 65.6075°W, 18–25.VI.2009, R. Webster & M.-A. Giguère, old red oak forest, Lindgren funnel trap (1, AFC). **York Co.**, New Maryland, off Hwy 2, E of Baker Brook, 45.8760°N, 66.6252°W, 26.IV.2005, R. P. Webster, old growth eastern white cedar swamp, in moss and litter at base of tree (1, RWC); Charters Settlement, 45.8267°N, 66.7343°W, 3.V.2006, R. P. Webster, *Carex* marsh, in litter and sphagnum (1, RWC); Mazerolle Settlement, 45.8729°N, 66.8311°W, 28.IV.2006, stream margin, in leaf litter at base of tree (1, RWC); 9 km W of Tracy off Rt. 645, 45.6889°N, 66.8002°W, 5.IV.2010, R. P. Webster, old beaver flowage, in grass litter on clay near small brook (1, RWC); 14 km WSW of Tracy, S of Rt. 645, 45.6603°N, 66.8607°W, 2.V.2010, R. P. Webster, black spruce bog, in sphagnum hummock with *Carex* and grasses (1, RWC); 15.5 km W of Tracy off Rt. 645, 45.6845°N, 66.8826°W, 10.V.2010, R. P. Webster, wet *Carex* marsh adjacent to old red pine forest, treading sphagnum (1, RWC).

**Map 18.**
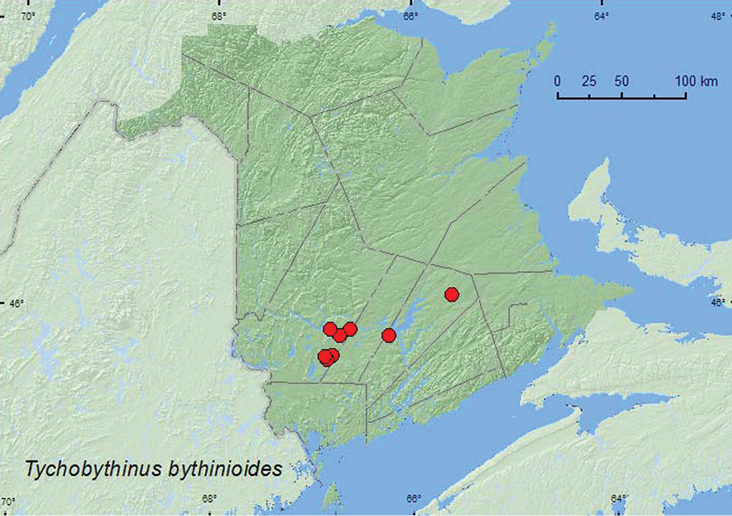
Collection localities in New Brunswick, Canada of *Tychobythinus bythinioides*.

##### Collection and habitat data.

In New Brunswick, this species was collected from a variety of wetland types. These include a tamarack bog, a black spruce bog, an old eastern white cedar swamp, *Carex* marshes, and an old beaver (*Castor canadensis* Kuhl) flowage with grasses. Adults occurred in sphagnum hummocks in bogs, in moss and litter at bases of trees, in litter and sphagnum in marshes, and in grass litter near a brook in an old beaver flowage. One individual was captured in a Lindgren funnel trap deployed in an old red oak forest. Chandler (1997) reports specimens being taken from sphagnum moss, swamp debris, tree holes, and from a mixture of birch/alder litter on the margin of a freshwater marsh. Adults were collected during April, May, and June.

##### Distribution in Canada and Alaska.

ON, QC, **NB**, NS (Davies 1991) [reported from NB by Majka et al. (2011) in error, C. Majka, personal communication].

### Tribe Tychini Raffray, 1904

#### Lucifotychus hirsutus

Chandler, 1991

http://species-id.net/wiki/Lucifotychus_hirsutus

Map 19

##### Material examined.

**New Brunswick, Restigouche Co.**, Berry Brook Protected Area (P.N.A.), 47.8140°N, 66.7578°W, 26.V.2007, R. P. Webster, old growth eastern white cedar swamp, in moss & leaf litter under alders (1, RWC).

**Map 19.**
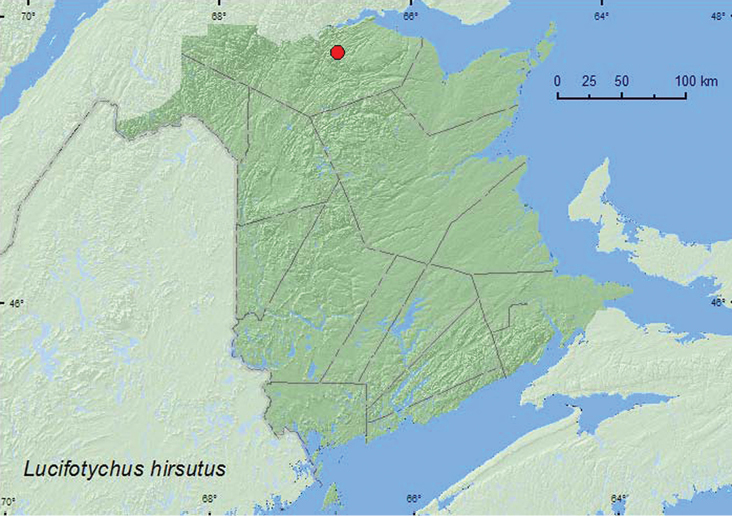
Collection localities in New Brunswick, Canada of *Lucifotychus hirsutus*.

##### Collection and habitat data.

The single adult from New Brunswick was collected in moss and leaf litter under alders in an old-growth eastern white cedar swamp during May. Chandler (1991) reported this species most commonly from conifer leaf and log litters. It has also been collected from mosses in Canada.

##### Distribution in Canada and Alaska.

MB, QC, **NB**, NS, NF (Chandler 1991).

### Supertribe Pselaphitae Latreille, 1802

**Tribe Ctenistini Blanchard, 1845**

#### Ctenisodes piceus

(LeConte, 1849)**

http://species-id.net/wiki/Ctenisodes_piceus

Map 20

##### Material examined. 

**New Brunswick, Carleton Co.**, Two Mile Brook Fen, 46.3619°N, 67.6733°W, 5.VIII.2004, 6.V.2005, M.-A. Giguère, R. P. Webster, & J. Edsall, old growth eastern white cedar swamp, in litter at base of cedar (2, RWC). **Northumberland Co.**, Goodfellow Brook P.N.A., 46.8943°N, 65.3796°W, 23.V.2007, R. P. Webster, old growth eastern white cedar swamp (many vernal pools), in litter, grasses and moss on hummocks near water (2, RWC). **Queens Co.**, Cranberry Lake P.N.A, 46.1125°N, 65.6075°W, 28.VII–6.VIII.2009, R. Webster & M.-A. Giguère, old red oak forest, Lindgren funnel trap (1, RWC). **York Co.**, 9 km W of Tracy off Rt. 645, 45.6889°N, 66.8002°W, 5.IV.2010, R. P. Webster, old beaver flowage, in grass litter on clay near small brook (1, RWC).

**Map 20.**
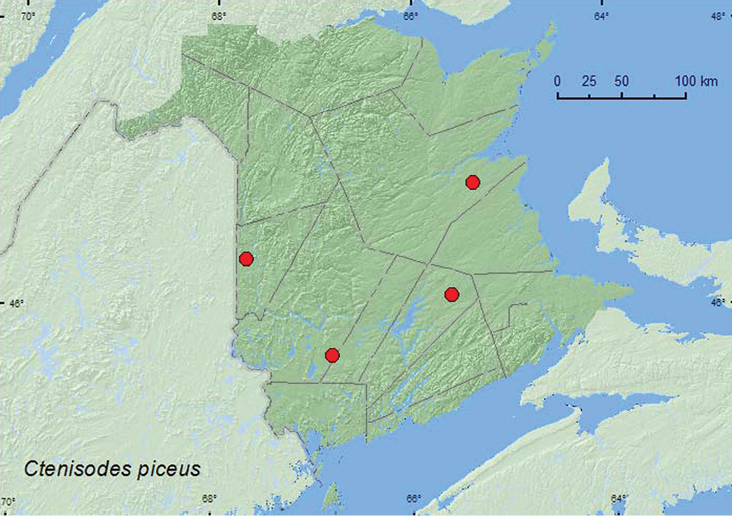
Collection localities in New Brunswick, Canada of *Ctenisodes piceus*.

##### Collection and habitat data.

In New Brunswick, *Ctenisodes piceus* was collected in old eastern white cedar swamps, an old red oak forest, and in an old beaver flowage. Adults were sifted from litter at the base of a cedar, from a mix of litter, grasses, and moss on hummocks (in eastern white cedar swamp), and from grass litter near a small brook. One adult was captured in a Lindgren funnel trap. Chandler (1997) reports specimens taken from leaf litter along the edges of streams and marshes, from mosses, from rotten wood, and at an ultraviolet light. Adults were collected during April, May, July, and August.

##### Distribution in Canada and Alaska.

ON, QC, **NB** (Davies 1991).

## Supplementary Material

XML Treatment for Euplectus acomanus

XML Treatment for Euplectus confluens

XML Treatment for Euplectus elongatus

XML Treatment for Pycnoplectus linearis

XML Treatment for Actiastes foveicollis

XML Treatment for Actiastes globiferum

XML Treatment for Bibloporus bicanalis

XML Treatment for Dalmosella tenuis

XML Treatment for Ramecia crinita

XML Treatment for Trimioplectus obsoletus

XML Treatment for Batrisodes frontalis

XML Treatment for Batrisodes riparius

XML Treatment for Batrisodes scabriceps

XML Treatment for Brachygluta luniger

XML Treatment for Decarthron abnorme

XML Treatment for Eutrichites zonatus

XML Treatment for Reichenbachia corporalis

XML Treatment for Tychobythinus bythinioides

XML Treatment for Lucifotychus hirsutus

XML Treatment for Ctenisodes piceus
